# Child physical abuse screening in a pediatric ED; Does TRAIN(ing) Help?

**DOI:** 10.1186/s12887-023-03927-0

**Published:** 2023-03-10

**Authors:** Theodore Heyming, Chloe Knudsen-Robbins, Supriya Sharma, Jonathan Thackeray, John Schomberg, Bryan Lara, Maxwell Wickens, Daphne Wong

**Affiliations:** 1 Children’s Health of Orange County, CHOC Hospital, 1201 W. La Veta Ave, Orange, CA 92868 USA; 2 Department of Emergency Medicine, University of California, Irvine, 3800 W. Chapman Ave, Suite 3200, Orange, CA 92868 USA; 3 University of Pittsburgh School of Medicine, 3550 Terrace St, Pittsburgh, PA 15213 USA; 4 Division of General Pediatrics, Harbor-UCLA Medical Center, 1000 W Carson St, Torrance, CA 90502 USA; 5 Department of Pediatrics, Dayton Children’s Hospital, 1 Childrens Plaza, Dayton, OH 45404 USA

**Keywords:** Pediatrics, Child physical abuse, Non-accidental trauma, Child maltreatment, Sentinel injury

## Abstract

**Background:**

Child maltreatment is distressingly prevalent yet remains under-recognized by healthcare providers. In 2015 the Ohio Children's Hospital Association developed the Timely Recognition of Abusive INjuries (TRAIN) collaborative in an effort to promote child physical abuse (CPA) screening. Our institution implemented the TRAIN initiative in 2019. The objective of this study was to examine the effects of the TRAIN initiative at this institution.

**Methods:**

In this retrospective chart review we recorded the incidence of sentinel injuries (SIS) in children presenting to the Emergency Department (ED) of an independent level 2 pediatric trauma center. SIS were defined and identified by a diagnosis of ecchymosis, contusion, fracture, head injury, intracranial hemorrhage, abdominal trauma, open wound, laceration, abrasion, oropharyngeal injury, genital injury, intoxication, or burn in a child < 6.01 months of age. Patients were stratified into pre-TRAIN (PRE), 1/2017–9/2018, or post-TRAIN (POST), 10/2019–7/2020, periods. Repeat injury was defined as a subsequent visit for any of the previously mentioned diagnoses within 12 months of the initial visit. Demographics/visit characteristics were analyzed using Chi square analysis, Fischer’s exact test, and student’s paired t-test.

**Results:**

In the PRE period, 12,812 ED visits were made by children < 6.01 months old; 2.8% of these visits were made by patients with SIS. In the POST period there were 5,372 ED visits, 2.6% involved SIS (*p* = .4). The rate of skeletal surveys performed on patients with SIS increased from 17.1% in the PRE period to 27.2% in the POST period (*p* = .01). The positivity rate of skeletal surveys in the PRE versus POST period was 18.9% and 26.3% respectively (*p* = .45). Repeat injury rates did not differ significantly in patients with SIS pre- versus post-TRAIN (*p* = .44).

**Conclusion:**

Implementation of TRAIN at this institution appears to be associated with increased skeletal survey rates.

**Supplementary Information:**

The online version contains supplementary material available at 10.1186/s12887-023-03927-0.

## Introduction

Child maltreatment remains a serious and pervasive problem; 1 in 7 children in the United States are thought to have been victims of child maltreatment within the past year, and approximately 5 children die each day as a direct result of abuse or neglect [1–3]. Beyond immediate physical harm, child maltreatment has been associated with increased risk for mental health disorders, cognitive/behavioral/developmental problems, substance use, suicide attempts, and lower levels of employment, education, and earnings [4–7]. The economic cost in the US alone is estimated to be $228 billion per year [8].

Despite these alarming statistics, child maltreatment, including physical/sexual/emotional abuse and neglect, remains under-reported and under-recognized [9–13]. Research has shown that sentinel injuries (SIS), although frequently overlooked by healthcare providers, must command further attention and evaluation as they may be the initial manifestation and presenting sign of child physical abuse (CPA) [14–21]. Additionally, studies have demonstrated that recurrent injury is not uncommon and that children with recurrent CPA are at increased risk of death [22–24]. The American Academy of Pediatrics has issued guidelines regarding not only the work-up of suspected abuse, including skeletal surveys, but also the specific physical exam findings concerning for abuse (which correspond to the diagnosis codes included in this study) [14]. The Emergency Department (ED) occupies an essential role in screening and evaluating child physical abuse given the large volume of children seen in EDs across the country each year as well as the fact that for some children the ED is their sole contact with medical professionals. Additionally, a majority of children attend non-pediatric EDs and thus many children are seen by providers without pediatric subspecialty training [25]. It is therefore important to identify effective training strategies for the unique ED environment.

In response, the Ohio Children's Hospital Association developed the Timely Recognition of Abusive INjuries (TRAIN) collaborative in 2015 to promote CPA screening and decrease missed SIS/recurrent CPA through provider education and standardized SIS protocols [26]. The group has since reported an approximately 50% increase in SIS identification and a 75% decrease in recurrent injury [26, 27]. However, current published literature is limited to a descriptive study of pre-TRAIN data and there appears to be no peer reviewed literature examining the effect of application of this program in the Emergency Department (ED) setting [28]. In October of 2019, our institution implemented the TRAIN initiative. This included a 2-h didactic session for nurses designed by a child maltreatment specialist with input from Ohio TRAIN champions. This training was followed by an online curriculum including quizzes; passing scores were required to complete training. Physicians/Nurse Practitioners/Physician Assistants participated in a 2-h didactic training session also designed by a child maltreatment specialist with input from Ohio TRAIN champions. This was followed by a question-and-answer session focusing on published child maltreatment literature and descriptive statistics. Marketing materials regarding CPA were also included as part of this intervention, and included signage at all workstations, in bathrooms, as well as on electronic boards in break rooms. Signage was developed by the TRAIN collaborative.

The objective of this study was two-fold–to describe the effects of the TRAIN initiative by examining pre and post implementation data as well as evaluate the transferability of the TRAIN initiative beyond its parent institutions. We aimed to describe our overall ED population and those with SIS pre and post TRAIN implementation to demonstrate overall characteristics and comparability. To evaluate the effects of TRAIN we aimed to examine the rate of skeletal surveys, the rate of positive skeletal surveys, repeat injuries, and social work/child protection team consults.

## Methods

In this retrospective chart review we recorded the incidence of SIS in children presenting to the ED of an independent, level 2 pediatric trauma center in the Southwestern United States. Patients were stratified into pre-TRAIN (PRE), 1/2017–9/2018, or post-TRAIN (POST), 10/2019–7/2020, periods, with a 12-month washout period between to allow for repeat injury analysis. Demographics and visit characteristics (including skeletal survey details, social work (SW) or child protection team (CPT) consults, International Classification of Diseases (ICD) codes) were collected by a team of trained data abstractors following an automated electronic health record query based on ICD-10 discharge diagnoses. Skeletal surveys were considered a proxy for CPA screening for several reasons, the most important being the necessity of ordering this study when concern for CPA was present, as well as its use throughout the CPA literature [9, 14, 15, 17]. This study was approved by this institution’s Institutional Review Board (#200,344).

### Definitions

SIS were defined and identified by a diagnosis of ecchymosis, contusion, fracture, head injury, intracranial hemorrhage, abdominal trauma, open wound, laceration, abrasion, oropharyngeal injury, genital injury, intoxication, or burn in a child < 6.01 months of age. A complete list of ICD-10 codes as well as diagnostic categories employed in the current study may be found in the supplementary materials. Children < 6.01 months of age (at initial visit) were included in this study. The age range and ICD diagnoses were chosen to correspond with those selected by the TRAIN collaborative [28].

A repeat injury was defined as a subsequent visit for any of the above-mentioned ICD diagnoses within 12 months of the initial visit. Follow-up visits for initial injuries were excluded.

A skeletal survey was considered positive if it demonstrated any fracture in the absence of initial imaging, if it demonstrated an additional fracture if initial ED imaging was obtained, or if the skeletal survey read recommended additional imaging and further imaging demonstrated an additional fracture.

### Statistical analysis

Chi square analysis was used to measure differences in demographics and visit characteristics in patients with SIS versus the general ED population and within the general ED population in the PRE and POST periods. Student's paired t-test was used to analyze mean age values. Fisher's exact test was used to compare demographics and visit characteristics between patients with SIS in the PRE and POST periods as well as those with repeat injuries. Rates of repeat injury were compared between PRE and POST TRAIN time periods using Student’s paired t-test. Visits for repeat injuries were removed from demographic analysis and repeat injury was set as a binary variable to prevent any single patient from contributing more than once. Logistic regression modeling was performed with skeletal survey or SW/CPT Consult set as outcome variables. Statistical analyses were performed using R, version 4.02.

## Results

In the PRE period, children < 6.01 months of age made 12,812 ED visits to the study institution; 2.8% of these visits were made by patients with SIS. In the POST period, there were 5,372 ED visits for children < 6.01 months old; 2.6%, were made by patients with SIS, *p* = 0.4.

### *Demographics of the general ED population* < *6.01 months, PRE vs POST*

In the PRE period 55.3% of patients in the general ED population were male compared to 53.3% in the POST period, *p* = 0.01. 63.4% of patients in the general ED population were white compared to 45.7% in the PRE versus POST period, *p* < 0.0001. In the PRE period 65.8% of patients were Hispanic compared to 67.4% in the POST period, *p* = 0.04. 18.8% of patients in the PRE period had private insurance compared to 24.1% in the POST period, *p* < 0.0001. Full demographics of patients in the general ED population < 6.01 months of age in the PRE versus POST periods can be found in Table 1.

**Table 1:** Demographics of the general ED population < 6.01 months of age, pre vs post-TRAIN^a^

	**General ED Pre-TRAIN (*n* = 12,448)**	**General ED Post-TRAIN (*n* = 5232)**	***p*-value**
**Sex**			0.01
Male	6888 (55.3%)	2789 (53.3%)	
Female	5560 (44.6%)	2443 (46.6%)	
**Race**			< .0001
White or Caucasian	7895 (63.4%)	2394 (45.7%)	
African American or Black	205 (1.64%)	91 (1.73%)	
Asian	782 (6.28%)	333 (6.36%)	
American Indian or Alaskan Native	20 (0.16%)	10 (0.19%)	
Native Hawaiian or Other Pacific Islander	48 (0.38%)	17 (0.32%)	
Unavailable or Unknown	3498 (28.1%)	2387 (45.6%)	
**Ethnicity**			0.04
Hispanic or Latino	8194 (65.8%)	3531 (67.4%)	
Non-Hispanic	4129 (33.1%)	1640 (31.3%)	
Unavailable or Unknown	125 (1.00%)	61 (1.16%)	
**Insurance**			< .0001
Private Insurance	2347 (18.8%)	1261 (24.1%)	
Public insurance	9568 (76.8%)	3838 (73.3%)	
Government (not Medicaid/Medicare)	80 (0.64%)	39 (0.74%)	
Self-Pay	414 (3.32%)	89 (1.70%)	
Other	39 (0.31%)	5 (0.09%)	

^a^ Excluding patients with sentinel injuries

### Demographics/visit characteristics of patients with SIS in the PRE vs POST periods

 In the PRE period 53.8% of patients with SIS were male compared to 65.1% of patients in the POST period (*p* = 0.04). A skeletal survey was performed on 17.1% versus 27.2% of patients in the PRE versus POST period (*p* = 0.01). There was a marginally statistically significant difference in reported race among patients with SIS in the PRE versus POST periods (*p* = 0.05). There was no statistically significant difference found with respect to age, ethnicity, insurance status, repeat injury, or ED SW/CPT consults. Complete demographics and visit characteristics of patients with SIS in the PRE and POST periods are included in Table 2.

**Table 2:** Demographics/visit characteristics of patients with SIS in Pre-TRAIN vs Post-TRAIN periods^a^

	**Patients with SIS pre-TRAIN (*n* = 327)**	**Patients with SIS post-TRAIN (*n* = 132)**	***p*-value**
**Age in months (mean, SD)**	4.04, 1.75	3.52, 1.72	0.5
**Gender**			0.04
Male	176 (53.8%)	86 (65.1%)	
Female	151 (46.1%)	46 (34.8%)	
**Race**			0.05^b^
White or Caucasian	185 (56.5%)	58 (43.9%)	
African American or Black	8 (2.44%)	4 (3.03%)	
Asian	39 (11.9%)	19 (14.3%)	
American Indian or Alaskan Native	1 (0.30%)	1 (0.75%)	
Unknown	94 (28.7%)	50 (37.8%)	
**Ethnicity**			0.62
Hispanic or Latino	166 (50.7%)	64 (48.4%)	
Non-Hispanic	156 (47.7%)	68 (51.5%)	
Unavailable or Unknown	5 (1.5%)	0(0%)	
**Insurance**			0.85
Private insurance	98 (29.9%)	43 (32.5%)	
Public insurance	217 (66.3%)	86 (65.1%)	
Other	1 (0.30%)	0 (0%)	
Government (not Medicaid/Medicare)	5 (1.52%)	2 (1.51%)	
Self-Pay	6 (1.83%)	1 (0.75%)	
**Skeletal Survey Performed**			0.01
Yes	56 (17.1%)	36 (27.2%)	
No	271 (82.8%)	96 (72.7%)	
**SW or CPT consult at ED visit**			0.89
Yes	63 (19.3%)	24 (18.2%)	
No	264 (80.7%)	108 (81.8%)	
**Repeat injury**			0.44
Patient with repeat injury	28 (8.56%)	8 (6.06%)	
Patient without repeat injury	299 (91.4%)	124 (93.9%)	

^a^Not including repeat visits

^b^Comparison of proportion of white to non-white patients

### *Demographics of patients with SIS vs the general ED population* < *6.01 months, PRE & POST*

In the PRE period, 51.9% of patients with SIS were male compared to 55.3% of the general ED population (*p* = 0.21), 55.2% were white compared to 63.4% (*p* < 0.0001), 53.5% were Hispanic compared to 65.8% (*p* < 0.0001), and 68.1% had public insurance compared to 76.8%, *p* = 0.0001. In the POST period 63.5% of SIS visits were made by males compared to 53.3% of the general ED population (*p* = 0.02), 45% were white compared to 45.7% (*p* = 0.01), and 49.2% were Hispanic compared to 67.4% (*p* < 0.0001). There was no statistically significant difference with respect to insurance status. Full demographic information for patients with SIS compared to the general ED population < 6.01 months of age in the PRE and POST periods can be found in Tables 3 and 4.

**Table 3:** Demographics of patients with SIS vs the general ED population < 6.01 months of age pre-TRAIN^a^

	**Patients with SIS Pre-TRAIN (*n* = 364)**	**General ED Pre-TRAIN (*n* = 12,448)**	***p*-value**
**Sex**			0.21
Male	189 (51.9%)	6888 (55.3%)	
Female	175 (48.0%)	5560 (44.6%)	
**Race**			< .0001
White or Caucasian	201 (55.2%)	7895 (63.4%)	
African American or Black	11 (3.02%)	205 (1.64%)	
Asian	43 (11.8%)	782 (6.28%)	
American Indian or Alaskan Native	1 (0.27%)	20 (0.16%)	
Native Hawaiian or Other Pacific Islander	0 (0%)	48 (0.38%)	
Unavailable or Unknown	108 (29.6%)	3498 (28.1%)	
**Ethnicity**			< .0001
Hispanic or Latino	195 (53.5%)	8194 (65.8%)	
Non-Hispanic	164 (45.0%)	4129 (33.1%)	
Unavailable or Unknown	5 (1.37%)	125 (1.00%)	
**Insurance**			0.0001
Private Insurance	103 (28.2%)	2347 (18.8%)	
Public insurance	248 (68.1%)	9568 (76.8%)	
Government (not Medicaid/Medicare)	5 (1.37%)	80 (0.64%)	
Self Pay	7 (1.92%)	414 (3.32%)	
Other	1 (0.27%)	39 (0.31%)	

^a^ Includes repeat visits

**Table 4:** Demographics of patients with SIS vs the general ED population < 6.01 months of age, post-TRAIN^a^

	**Patients with SIS Post-TRAIN (*n* = 140)**	**General ED Post-TRAIN (*n* = 5,232)**	***p*-value**
**Sex**			0.02
Male	89 (63.5%)	2789 (53.3%)	
Female	51 (36.4%)	2443 (46.6%)	
**Race**			0.01
White or Caucasian	63 (45%)	2394 (45.7%)	
African American or Black	4 (2.85%)	91 (1.73%)	
Asian	20 (14.2%)	333 (6.36%)	
American Indian or Alaskan Native	1 (0.71%)	10 (0.19%)	
Native Hawaiian or Other Pacific Islander	0 (0%)	17 (0.32%)	
Unavailable or Unknown	52 (37.1%)	2387 (45.6%)	
**Ethnicity**			< .0001
Hispanic or Latino	69 (49.2%)	3531 (67.4%)	
Non-Hispanic	71 (50.7%)	1640 (31.3%)	
Unavailable or Unknown	0 (0%)	61 (1.16%)	
**Insurance**			0.22
Private Insurance	44 (31.4%)	1261 (24.1%)	
Public insurance	93 (66.4%)	3838 (73.3%)	
Government (not Medicaid/Medicare)	2 (1.42%)	39 (0.74%)	
Self Pay	1 (0.71%)	89 (1.70%)	
Other	0 (0%)	5 (0.09%)	

^a^ Includes repeat visits

### Demographics/visit characteristics of patients with & without repeat injuries

Among patients with SIS, 36 patients had at least 1 repeat injury, 423 had no repeat injuries. Female patients accounted for 58.3% of patients with repeat injury as compared to 41.4% of patients without repeat injury, *p* = 0.05, and 72.2% of patients with repeat injury were Hispanic compared to 48.1% of those without repeat injury, *p* = 0.04. There was no statistically significant difference found with respect to age, insurance, skeletal surveys, or SW/CPT consult. In the PRE period 2.1% of patients with SIS had > 2 repeat injuries compared to 0% in the POST period (*p* = 0.2). Demographics and visit characteristics for patients with and without repeat injury are included in Table 5.

**Table 5:** Demographics/visit characteristics of patients with/without repeat injuries

	**Patients with repeat injury (*n* = 36)**	**Patients without repeat injury (*n* = 423)**	***p*-value**
**Age (mean, SD)**	3.7, 1.7	3.4, 1.7	0.28
**Gender**			0.05
Male	15 (41.6%)	247 (58.5%)	
Female	21 (58.3%)	176 (41.4%)	
**Race**			0.68
White	19 (52.7%)	224 (52.8%)	
African American	2 (5.55%)	10 (2.36%)	
Asian	4 (11.1%)	54 (12.7%)	
American Indian	0 (0%)	2 (0.47%)	
Pacific Islander	0 (0%)	0 (0%)	
Unknown	11 (30.5%)	133 (31.5%)	
**Ethnicity**			0.04
Hispanic	26 (72.2%)	204 (48.1%)	
Non-Hispanic	10 (27.7%)	214 (50.7%)	
Unknown	0 (0%)	5 (1.18%)	
**Insurance**			0.17
Private Insurance	5 (13.8%)	136 (32.2%)	
Public insurance	30 (83.3%)	273 (64.4%)	
Other	0 (0%)	1 (0.23%)	
Government (not Medicaid/Medicare)	0 (0%)	7 (1.65%)	
Self Pay	1 (2.77%)	6 (1.42%)	
**Skeletal Survey Performed**^**a**^			0.19
Yes	4 (11.1%)	88 (20.8%)	
No	32 (88.8%)	335 (79.1%)	
**SW/CPT consult**			0.27
Yes	4 (11.1%)	83 (19.6%)	
No	32 (88.8%)	340 (80.3%)	
**ICD-10 Category**^**b**^			0.0004
Open wounds/abrasion/laceration	3 (8.33%)	18 (4.2%)	
Contusion/ecchymosis (excluding head)	1 (2.77%)	20 (4.7%)	
Contusion/ecchymosis to head/face	6 (16.6%)	61 (14.4%)	
Fractures (excluding skull fractures)	1 (2.77%)	38 (8.9%)	
Major head Injury	3 (8.33%)	55 (13.0%)	
Minor head injury	24 (66.6%)	235 (55.5%)	
Burns	1 (2.77%)	9 (2.1%)	
Genital injury	0 (0%)	4 (0.9%)	
Oropharyngeal injury	1 (2.77%)	0 (0%)	
Foreign Body	0 (0%)	2 (0.4%)	
Other	0 (0%)	2 (0.4%)	

^a^Reflecting ED skeletal surveys at initial visit

^b^Excludes ICD codes of child abuse

### Skeletal surveys, ICD diagnoses, and positivity rates

 In the PRE period, 18.9% of skeletal surveys in patients with SIS who received skeletal surveys in the ED were positive. In the POST period, 26.3% were positive (*p* = 0.45). The percent of patients with skeletal surveys stratified by ICD diagnosis category as well as positivity rates may be found in Tables 6 and 7.

**Table 6:** Skeletal Survey performance and SW/CPT consult by diagnosis category for pre-TRAIN and Post-TRAIN patients^a^

Diagnosis category	Skeletal Survey Performed (*n* = 96)	Skeletal Survey Not Performed (*n* = 408)	SW/CPT consult (*n* = 88)
Open wounds/abrasion/laceration	0 (0%)	23 (100%)	0 (0%)
Contusion/ecchymosis (excluding head)	3 (14.2%)	18 (85.7%)	2 (9.52%)
Contusion/ecchymosis to head/face	18 (24.3%)	56 (75.6%)	15 (20.2%)
Fractures (excluding skull fractures)	23 (53.4%)	19 (45.23%)^b^	20 (47.6%)
Major head injury	48 (78.6%)	13 (21.3%)^c^	28 (45.9%)
Minor head injury	18 (6.18%)	274 (93.8%)	22 (7.53%)
Burns	2 (20%)	8 (80%)	6 (60%)
Genital injury	0 (0%)	4 (100%)	2 (50%)
Oropharyngeal injury	1 (100%)	0 (0%)	1 (100%)
Foreign Body	0 (0%)	1 (100%)	0 (0%)
Other	1 (50%)	1 (50%)	1 (50%)

^a^This table represents the skeletal survey performed in instances of both injury and repeat injury, ICD categories are not mutually exclusive

^b^Of patients with a diagnosis of fracture and no skeletal survey, 70% were clavicle fractures or toe fractures

^c^Of patients with a major head injury and no skeletal survey, 50% had a SW/CPT consult or were admitted

**Table 7:** Skeletal survey results by diagnosis category for pre-TRAIN and post-TRAIN patients^a^

Diagnosis category	Skeletal Survey Normal (*n* = 74)	Skeletal Survey Positive (*n* = 22)
Open wounds/abrasion/laceration	NA	NA
Contusion/ecchymosis (excluding head)	3 (100%)	0 (0%)
Contusion/ecchymosis to head/face	16 (88.8%)	2 (11.1%)
Fractures (excluding skull fractures)	9 (39.1%)	14 (60.8%)
Major head injury	42 (87.5%)	6 (12.5%)
Minor head injury	17 (94.4%)	1 (5.5%)
Burns	2 (100%)	0 (0%)
Genital injury	NA	NA
Oropharyngeal injury	1 (100%)	0 (0%)
Foreign Body	NA	NA
Other	1 (100%)	0 (0%)

^a^This table represents the skeletal survey performed in instances of both injury and repeat injury, ICD categories are not mutually exclusive

### Logistic regression models

Hispanic ethnicity was associated with statistically significantly decreased odds of a skeletal survey (OR = 0.89, *p* = 0.005). The post-TRAIN phase was associated with statistically significantly increased odds of a skeletal survey (OR = 1.14, *p* = 0.002). Age was associated with decreased odds of SW/CPT consult, as was Hispanic ethnicity (OR = 0.98, *p* = 0.004; OR = 0.90, *p* = 0.01 respectively). Table 8 includes OR for logistic regression modelling with skeletal survey set as the outcome variable. Table 9 includes OR for logistic regression modelling with SW/CPT consult set as the outcome variable.

**Table 8:** Logistic regression model for prediction of skeletal survey

	**OR**	**95% Confidence Interval**	***P*-value**
**Age**	0.99	0.97 – 1.00	0.07
**Sex (Female reference = Female)**			
Male	1.02	0.95 – 1.10	0.56
**Race (Reference = Non-White)**			
White	1.01	0.94 – 1.09	0.73
**Ethnicity (Reference = non-Hispanic)**			
Hispanic	0.89	0.82 – 0.97	0.005
**Insurance**			
Medicaid	1.02	0.94 – 1.11	0.64
**TRAIN Phase**			
POST- TRAIN	1.14	1.05 – 1.24	0.002

**Table 9:** Logistic regression model for prediction of SW/CPT consult

	**OR**	**95% Confidence Interval**	***P*-value**
**Age**	0.98	0.97 – 0.99	0.004
**Sex (Female reference = Female)**			
Male	1.04	0.96 – 1.11	0.33
**Race (Reference = Non-White)**			
White	1.02	0.95 – 1.10	0.62
**Ethnicity (Reference = non-Hispanic)**			
Hispanic	0.90	0.84 – 0.98	0.01
**Insurance**			
Medicaid	1.09	1.00 – 1.19	0.05
**TRAIN Phase**			
POST- TRAIN	1.02	0.95 – 1.11	0.55

## Discussion

The authors sought to describe effects associated with the TRAIN initiative by examining pre- and post- implementation data as well as to evaluate the transferability of the TRAIN initiative to our study institution. We observed 1) changes in overall skeletal survey rates in the pre to post TRAIN periods, 2) opportunity for improvement with respect to skeletal surveys for specific ICD diagnoses, 3) stability of skeletal survey positivity rates, 4) changes in general ED demographics in the pre- and post-TRAIN period compared to those with SIS, 5) stability in SIS rates, and 6) stability in repeat injury rates. These observations are compared to existing literature on screening for CPA in EDs.

### Overall skeletal survey rates

Following the implementation of the TRAIN initiative we found a statistically significant increase in the rate of skeletal surveys conducted in children with SIS. Logistic regression modeling also demonstrated increased OR of skeletal surveys in the post-TRAIN phase. This suggests that the involved didactics and marketing materials may indeed spur enhanced examination of the types of injuries that are possible indicators of CPA.

### ICD diagnosis and skeletal surveys

The overall skeletal survey rate in children with bruising or major head injuries appear within ranges reported by Lindberg et al. as well as by the Ohio TRAIN investigators [17, 28]. Crumm et al. reported significantly higher rates of skeletal surveys in children with bruises though this followed the development of a high-risk bruising screening pathway [15]. A considerable portion of patients in the current study had sentinel injury diagnoses of fracture alone. Given the limitations of this study the impetus for obtaining an initial x-ray or skeletal survey was not always clear. It is possible that historical or physical exam findings such as bruising may have triggered further evaluation. Given the intrinsically fast-paced nature of the ED as well as continued ED overcrowding, ED physicians tend not to include ICD diagnosis for all physical exam findings. This may have altered the rate of observed skeletal surveys in certain types of injuries. Overall, our data suggests that there remains significant opportunity for improvement with respect to skeletal survey evaluation as 45% of patients with a diagnosis of fracture and 24% of patients with a diagnosis of a major head injury did not receive skeletal surveys. Of note, 70% of patients with a diagnosis of fracture and no skeletal survey had clavicle or toe fractures, and 50% of those with major head injury and no skeletal survey had a SW/CPT consult or were admitted.

### Skeletal survey positivity rates

The skeletal survey positivity rate in this study remained stable; this is reassuring as it suggests that heightened attention does not inevitably increase potentially unnecessary radiation exposure through excess skeletal surveys. Additionally, a stable positivity rate in conjunction with increasing skeletal survey rates also suggests possible higher rates of missed CPA prior to the TRAIN initiative. Changing patterns of ED use and child maltreatment during the COVID-19 pandemic may have also contributed to this finding [29]. Notably, positivity rates in this study appear similar to those seen in other investigations [9, 30–33].

### SW/CPT consults

There was no significant difference in SW/CPT consults in the post-TRAIN period. We did not assess for confirmed maltreatment in this study thus could not examine rates of substantiated CPA.

### Demographics and SIS in the PRE/POST periods

Demographic trends proved difficult to assess given the overall demographic shifts seen in the general ED population throughout the course of the study. Insurance status of patients with SIS in the PRE period were significantly different than that of the general ED population. In the POST period, there was no significant difference in insurance status between patients with SIS compared to the general ED population suggesting improved universal screening with respect to socio-economic status (SES). Additionally, it appears that the percent of patients reporting white race in the POST period more closely mirrors that of the general ED population. The current findings may reflect increased provider awareness of inherent biases following the TRAIN initiative. Alternatively, it is possible that these findings suggest no increased recognition of SIS as patients with SIS in the PRE period were more likely to be non-white and the percent of non-white patients increased in the POST period. Studies have shown that CPA screening not universally applied more often targets minorities and those of lower SES backgrounds [14, 31, 34, 35].

Gender was associated with a significant difference between patients with SIS in the PRE versus POST periods, as a higher proportion of patients in the POST period were males. Although males represented a higher proportion of patients than females in the PRE period as well, this was consistent with the general ED population. This data may indicate improved SIS recognition in the POST period as male gender has been associated with increased risk of physical abuse [2, 17, 31, 36–38]. Strikingly, male gender has been found to be an independent predictor of missed diagnosis of abuse and males have a higher incidence of death from abuse than females [3, 12]. However, previous studies did not report findings associated with physical abuse in the context of general ED demographics. They also typically included wider age ranges than in the current study or relied on self-reported statistics. Further research may help elucidate if male infants are at increased risk for CPA, and if so, what interventions to address this risk may be feasible.

### Sentinel injury rates

The SIS rate remained stable during the PRE and POST periods, at a level approximately 3.5-fold greater than that reported by Lindberg et al. in a study including patients < 12 months of age at several pediatric tertiary care center Emergency Departments [17]. This difference may reflect the current study's broader definition of SIS, which included unspecified head injuries, lacerations, and abrasions.

### Repeat injuries

There was no significant difference between PRE and POST periods with respect to rates of repeat injury. Of note, 2.1% of patients in the PRE period had > 2 repeat injuries compared to 0% in the POST period; however, this finding was not statistically significant. The stability of the SIS and repeat injury rates observed in this study contrast to the approximately 50% increase in SIS identification and nearly 75% reduction in repeat injury reported by the multi-center Ohio TRAIN collaborative [26, 27]. It is striking that the SIS rate did not increase as expected or as observed in Ohio. This may be due in part to two main differences in TRAIN implementation at the study institutions. At the current study institution skeletal surveys were ordered at the discretion of the physician, and ICD diagnoses of physical exam findings were not required to be included in the discharge diagnosis. Although it is difficult to assess guideline adherence in the multi-center study in Ohio given the lack of published literature, it is possible that allowing for provider discretion at our institution may have negated some effects of anti-bias training, and that patients were not correctly identified due to potential missing ICD diagnoses. Moreover, the patient population in this study may differ significantly from the population seen in Ohio, as recognized maltreatment rates are known to vary by state and ethnic background [3]. However, it is difficult to directly compare SIS rate results with the original multi-center TRAIN study given the unknown inter-study differences including study inclusion criteria/SIS identification and institution type. It is possible that in the current study the steady SIS rate contributed to the stable repeat injury rates. Additionally, we tracked patients only at a single institution, potentially limiting our ability to detect repeat injury though the reported repeat injury rates in the current study are similar to those reported by the TRAIN collective after the development of TRAIN [28].

Analysis also revealed significant differences in ethnicity and marginally significant differences in gender between patients with versus without repeat injury. There was no statistically significant difference in the rates of skeletal surveys done at the initial ED visit in patients with and without repeat injury. Hispanic ethnicity was more common in patients with repeat injury, but less common among all patients with SIS as compared to the general ED population (in both the PRE and POST periods). The underlying cause of this finding is unclear. Although a higher proportion of patients with SIS were male, a higher percentage of patients with repeat injury were female. Within the bounds of this study, it is difficult to assess if this represents provider failure to properly evaluate initial injuries. It is possible that the higher rates of repeat injury visits demonstrated in female children reflect either increased or decreased parental concern for female infants [23]. Deans et al. did not identify gender as a predictive factor for repeat injury; however, their study cohort included only children with diagnoses of maltreatment or skeletal surveys [23].

### Limitations

Importantly, inclusion in the current study was based solely on a discharge diagnosis of SIS; chief complaint and physical exam findings were not considered. This may have affected both the skeletal survey rate as well as the size of the overall study population. Of note, there are several pediatric hospitals within 100 miles of the current study institution; it is possible the patient population seen at our institution differs significantly from those seen at institutions serving other catchment areas. Other limitations of this study include a relatively small sample size, especially with respect to the POST and repeat injury data, which may have underpowered our ability to detect significant differences. The COVID-19 pandemic may have effected ED use and child maltreatment in the POST period as well; there was a documented decline in pediatric ED attendance during the pandemic and several studies have documented various changes with respect to child maltreatment and the presentations of child maltreatment during the pandemic [39–42]. A potential confounder of this study was the implementation of an electronic health record tool/trigger based-alert system to aid in the identification of child maltreatment (the Child Abuse Clinical Decision Support System) on July 15^th^, 2020 (during the POST washout period – the 12 month follow-up period for patients initially seen during the POST period). This may have contributed to increased repeat injury recognition although repeat injury rates did not appear significantly different. In addition, there was a large percentage of patients with unknown race which may have skewed racial demographic statistics.

## Conclusion

Implementation of TRAIN at this institution appears to be associated with increased skeletal survey rates. The increased skeletal survey rate in conjunction with a stable positivity rate suggests increased identification of possible child physical abuse following the TRAIN initiative. Although this study did not demonstrate an increase in sentinel injury identification or reduction in repeat injury as reported by the original TRAIN project, application of this initiative at this institution appears to be associated with increased evaluation of patients with sentinel injuries.

## Supplementary Information

**Additional file 1.**

**Additional file 2.**

## Data Availability

The datasets used and/or analyzed during the current study are available from the corresponding author on reasonable request.
