# Supplementary material for: Child physical abuse screening in a pediatric ED; Does TRAIN(ing) Help?
Source: BMC Pediatr. 2023 Mar 10;23:117. doi: 10.1186/s12887-023-03927-0 (PMC9998251; doi:10.1186/s12887-023-03927-0)
Supplement: Supplementary file 1 — Additional file 1. [file 12887_2023_3927_MOESM1_ESM.docx]

COVID-19 - Coronavirus Disease 2019

CPA - child physical abuse

CPT - child protection team

ED - Emergency Department

ICD - International Classification of Diseases

NAT - non-accidental trauma

POST - post-TRAIN

PRE - pre-TRAIN

SIS - sentinel injuries

SW - social work

TRAIN - Timely Recognition of Abusive INjuries
