# Supplementary material for: Child physical abuse screening in a pediatric ED; Does TRAIN(ing) Help?
Source: BMC Pediatr. 2023 Mar 10;23:117. doi: 10.1186/s12887-023-03927-0 (PMC9998251; doi:10.1186/s12887-023-03927-0)
Supplement: Supplementary file 2 — Additional file 2. [file 12887_2023_3927_MOESM2_ESM.docx]

**Sentinel injury ICD-10 codes:**

R23.3

S02.0XXA-S02.92xS

S12.000A-S12.691S

S12.8xxA-S12.8xxS

S12.9xxA-S12.9xxS

S22.000A-S22.9xxS

S32.000A-S32.9xxS

S42.001A-S42.92x

S52.001A-S52.92xS

S62.001A-S62.92xS

S72.001A-S72.92xS

S82.001A-S82.92xS

S92.001A-S92.919S

S06.360A

I62.9

S06.310A-S06.389S

S06.4X0A-S06.6X0S

S06.300A- S06.309S

S06.810A-S06.819S

S06.890A-S06.9X9S

S36.200A-S36.69XS

S36.112A-S36.119S

S36.00XA-S36.09XS

S37.001A-S37.099A

S37.10XA-S37.99XS

S36.122A-S36.13XS

S36.81XA-S36.99XS

S37.812A-S37.819S

S37.892A-S37.99XS

S36.99XA-S36.99XS

S37.99XA-S37.99XS

S01.101A-S01.159S

S05.40XA-S05.42XS

S05.20XA-S05.72XS

S01.311A-S01.319S

S08.111A-S08.129S

S09.20XA-S09.991S

S01.00XA-S01.05XS

S01.20XA-S01.25XS

S01.401A-S01.95XS

S08.0XXA-S08.89XS

S11.011A-S11.95XS

S21.101A-S21.159S

S21.201A-S21.259S

S31.000A-S31.050S

S31.801A-S31.829S

S31.20XA-S31.552S

S21.001A-S21.059S

S31.100A-S31.159S

S41.001A-S41.159S

S46.021A-S46.029S

S46.121A-S46.129S

S46.221A-S46.229S

S46.321A-S46.329S

S46.821A-S46.829S

S46.921A-S46.929S

S51.001A-S51.859S

S61.501A-S61.559S

S56.021A-S56.029S

S56.121A-S56.129S

S56.221A-S56.229S

S56.321A-S56.329S

S56.421A-S56.429S

S56.521A-S56.529S

S56.821A-S56.829S

S56.921A-S56.929S

S61.401A-S61.459S

S66.821A-S66.929S

S61.001A-S61.359S

S66.021A-S66.029S

S66.120A-S66.129S

S66.221A-S66.229S

S66.320A-S66.329S

S66.421A-S66.429S

S66.520A-S66.529S

S41.001A-S41.009S

S41.101A-S41.109S

S51.001A-S51.009S

S51.801A-S51.809S

S61.009A-S61.009S

S61.101A-S61.109S

S61.200A-S61.209S

S61.300A-S61.309S

S61.401A-S61.409S

S61.501A-S61.509S

S68.011A-S68.029S

S68.511A-S68.529S

S68.110A-S68.129S

S68.610A-S68.629S

S68.411A-c8.429S

S68.711A-S68.729S

S71.001A-S71.059S

S71.101A-S71.159S

S76.021A-S76.029S

S76.121A-S76.129S

S76.221A-S76.229S

S76.321A-S76.329S

S76.821A-S76.829S

S76.929A-S76.929S

S81.001A-S81.059S

S81.801A-S81.859S

S91.001A-S91.059S

S86.021A-S86.029S

S86.121A-S86.129S

S86.221A-S86.229S

S86.321A-S86.329S

S86.821A-S86.829S

S86.921A-S86.929S

S96.211A-S96.229S

S96.821A-S96.829S

S96.921A-S96.929S

S91.301A-S91.359S

S96.221A-S96.229S

S96.821A-S96.829S

S96.921A-S96.929S

S91.101A-S91.159S

S91.201A-S91.259S

S96.021A-S96.129S

S71.001A-S71.109S

S81.001A-S81.009S

S81.801A-S81.809S

S98.111A-S98.149S

S98.211A-S98.229S

S98.011A-S98.029S

S98.311A-S98.329S

S98.911A-S98.929S

S78.011A-S78.929S

S88.011A-S88.929S

S01.502A-S01.552S

S02.5XXA-S02.5XXS

S00.00XA-S00.02XS

S00.05XA-S00.07XS

S00.30XA-S00.32XS

S00.34XA-S00.37XS

S00.401A-S00.429S

S00.441A-S00.479S

S00.501A-S00.522S

S00.541A-S00.572S

S00.80XA-S00.82XS

S00.84XA-S00.87XS

S00.90XA-S00.92XS

S00.94XA-S00.97XS

S10.10XA-S10.17XS

S10.80XA-S10.82XS

S10.84XA-S10.87XS

S10.90XA-S10.92XS

S10.94XA-S10.97XS

S20.101A-S20.179S

S20.301A-S20.479S

S20.90XA-S20.97XS

S30.810A-S30.98XS

S40.211A-S40.929S

S50.311A-S50.919S

S60.811A-S60.919S

S60.511A-S60.579S

S60.921A-S60.929S

S60.311A-S60.479S

S60.931A-S60.949S

S70.211A-S70.929S

S80.211A-S80.929S

S90.511A-S90.579S

S90.911A-S90.919S

S90.411A-S90.476S

S90.811A-S90.879S

S90.921A-S90.936S

T07

S00.03XA-S00.03XS

S00.33XA-S00.33XS

S00.431A-S00.439S

S00.531A-S00.532S

S00.83XA-S00.83XS

S00.93XA-S00.93XS

S10.0XXA-S10.0XXS

S10.83XA-S10.83XS

S10.93XA-S10.93XS

S00.10XA-S00.12XS

S05.10XA-S05.12XS

S20.00XA-S20.02XS

S20.20XA-S30.3XXS

S40.011A-S40.029S

S50.00XA-S50.12XS

S60.00XA-S60.059S

S60.10XA-S60.229S

S70.00XA-S70.12XS

S80.00XA-S80.12XA

S90.00XA-S90.129S

S90.211A-S90.219S

S90.221A-S90.229S

S90.30XA-S90.32XS

S30.201A-S30.202S

S30.21XA-S30.23XS

S31.40XA-S31.551S

S38.211A-S38.232S

T26.00XA-T26.92XS

T20.00XA-T20.79XS or T20

T28.411A-T28.419S

T28.911A-T28.919S

T21.00XA-T21.79XS

T22.00XA-T22.799S

T23.001A-T23.799S

T24.001A-T25.799S

T30.0

T27.0XXA-T28.99XS

T31.0-T32.99XS

T30.4

**Excluded:**

H11.30-H11.33 (for <0.5 months only)

S00.201A-S00.279S

V02.00XA-V09.9XXS

V12.0XXA-V79.9XXS

V80.31XA-V80.52XA

V81.0XXA-V81.1XXS

V82.0XXA-V82.1XXS

V84.0XXA-V89.9XXS

S00.06XA-S00.06XS

S00.16XA-S00.16XS

S10.86XA-S10.86XS

S00.16XA-S00.16XS

S10.86XA-S10.86XS

S20.161A-S20.169S

S20.361A-S20.369S

S20.461A-S20.469S

S20.96XA-S20.96XS

S30.860A-S30.867S

S40.261A-S40.269S

S40.861A-S40.869S

S50.361A-S50.369S

S50.861A-S50.869S

S60.361A-S60.369S

S60.460A-S60.469S

S60.561A-S60.569S

S60.861A-S60.869

W53.01XA-W59.89XS

W61.01XA-W62.9XXS

T63.001A-T63.94XS

T63.001A-T63.94XS

S30.842A-S30.842S

S60.341A-S60.349S

S60.440A-S60.449S

S90.441A-S90.446S

Y92.230-Y92.239

P10.0-P15.9

**ICD-10 Diagnoses categories (for ICD diagnoses found in this study):**

*Open wounds/abrasion/lacerations*

S30.810A

S50.812A

S60.419A

S60.429A

S61.009A

S61.012A

S61.209A

S61.219A

S61.409A

S61.419A

S80.822A

S91.209A

*Fractures (excluding skull fractures)*

S22.39XA

S22.39XD

S22.42XA

S22.49XA

S22.49XD

S42.001A

S42.001D

S42.002A

S42.009A

S42.302A

S42.309A

S42.411A

S42.413A

S42.416A

S52.331A

S52.90XA

S52.91XA

S52.92XA

S72.322A

S72.402A

S72.90XA

S72.91XA

S72.92XA

S82.102A

S82.201A

S82.209A

S82.90XA

S92.403A

*Major head injury (excludes bruising/abrasions etc. to face, includes intracranial injury/ skull fractures etc.)*

S02.0XXA

S02.119A

S02.80XA

S02.81XA

S02.85XA

S02.91XA

S06.360A

S06.4X9A

S06.5X0A

S06.5X9

S06.6X0A

S06.6X9A

S06.9X9A

*Minor head injury*

S09.90XA

S09.93XA

S09.91XA

S00.01XA

S09.8XXA

S09.90XA

S09.92XA

S00.31XA

S00.32XA

S00.411A

S00.419A

S00.81XA

S01.01XA

S01.112A

S01.119A

S01.311A

S01.81XA

S01.85XA

S01.501A

S01.511A

S00.521A

S06.0X9A

*Oropharyngeal injury*

S01.512A

*Genital injury*

S30.21XA

S30.812A

T21.27XA

*Burns*

T21.01XA

T22.239A

T24.009

T24.009A

T24.202A

T25.232A

T30.0

*Foreign body*

S00.452A

S00.459A

*Ecchymosis/contusion to head*

S00.03XA

S00.03X

S00.83XA

S00.93XA

S00.12XA

S00.33XA

S00.531A

H11.30

*Ecchymosis/contusion to body other than head*

R23.3

S30.0XXA

S30.1XXA

S40.019A

S40.022A

S50.11XA

S60.00XA

S60.222A

S80.10XA

S90.00XA

S90.129A

S90.31XA

S90.32XA

*Child physical abuse-confirmed/Child physical abuse-suspected/Shaken infant syndrome*

T74.12XA

T74.4XXA

T74.92XA

T76.12XA

T76.92XA

*Other:*

F15.929

T14.8XXA
